# Commentary on: Is preheat necessary for the measurement of 8‐oxo‐7,8‐dihydroguanosine and 8‐oxo‐7,8‐dihydro‐2′‐deoxyguanosine in urine samples

**DOI:** 10.1002/jcla.24874

**Published:** 2023-04-01

**Authors:** Chiung‐Wen Hu, Marcus S. Cooke, Yi‐Jhen Wang, Mu‐Rong Chao

**Affiliations:** ^1^ Department of Public Health Chung Shan Medical University Taichung 402 Taiwan; ^2^ Oxidative Stress Group, Department of Cell Biology, Microbiology and Molecular Biology University of South Florida Tampa Florida 33620 USA; ^3^ Department of Occupational Safety and Health Chung Shan Medical University Taichung 402 Taiwan; ^4^ Department of Occupational Medicine Chung Shan Medical University Hospital Taichung 402 Taiwan

**Keywords:** biomarkers, DNA adducts, oxidative stress, precipitates, urine

We read with great interest the published paper by Zou et al.^1^ In this paper, they investigated whether “preheating” is necessary for the accurate measurement of two urinary biomarkers of oxidative stress 8‐oxo‐7,8‐dihydroguanosine (8‐oxoG) and 8‐oxo‐7,8‐dihydro‐2′‐deoxyguanosine (8‐oxodG),^2^ using an LC‐MS/MS method. In the abstract, they concluded that it is not always necessary to preheat frozen urine samples to release 8‐oxoG and 8‐oxodG from precipitates that are often present. However, we are concerned that this statement may mislead readers because warming previously frozen urine samples is the best strategy to ensure the accuracy and consistency of 8‐oxoG and 8‐oxodG analysis. Indeed, because these precipitates may contain compounds of interest, if the precipitates are not properly redissolved, this may generate erroneous data. Therefore, we wish to clarify a number of points and provide some suggestions as to good practices when analyzing DNA adducts in urine.

1. Redissolving any precipitates in urine is critical to releasing any associated adducts. Therefore, the vigorous shaking of the samples after a complete thaw (e.g., warming to 37°C) to re‐dissolve these precipitates before analyses is essential.^3^, ^4^, ^5^ The authors reported that thawing the urine samples at room temperature (RT) for 30 or 120 min or at 37°C for 15 or 90 min did not affect the determination of 8‐oxoG and 8‐oxodG. This is because the precipitates in urine had been completely redissolved by the authors “diluting the urine 11 times with an aqueous solution” after thawing. The dilution process contributed to the release of 8‐oxoG and 8‐oxodG from the precipitates. Therefore, in their study, no significant difference was found for the urinary 8‐oxoG and 8‐oxodG results between thawing at RT and 37°C. The authors, therefore, conclude, incorrectly, that preheating is not necessary.
2. Thorough mixing of the precipitates in urine after thawing is unlikely to guarantee that each aliquot (only 0.01 mL) has a homogeneous distribution of precipitates in suspension. The authors stated that they thoroughly mixed the urine samples after thawing without discarding precipitates. We have demonstrated that after vortexing, precipitates may be initially uniformly distributed in suspension, but this lasts less than 3 s, and they settle to the bottom of the tube within 10 s (Figure 1). Therefore, vortexing/mixing is not sufficient in order to obtain reliable and reproducible results. It is difficult to obtain an aliquot containing a “representative” amount of precipitates within only a few seconds, and this also depends very much on the operator's experience.
3. Our suggestions for dealing with the precipitates in urine. In addition to the targeted analysis of specific DNA adducts in urine, the study of the totality of DNA adducts in urine (i.e., urinary DNA adductomics)^6^ is an emerging approach for human biomonitoring. To accurately measure DNA modifications/adducts in urine, it is preferred to analyze the fresh urines, which are without precipitates. However, in most cases, urines are likely to have been collected and stored at a low temperature (<−20°C) before analysis, which frequently results in the formation of precipitates. To comprehensively address the urinary precipitates, we provide two strategies: (i) when fresh urine is obtained, it can be divided immediately into small aliquots (e.g., 0.1 mL) prior to storage. It is important that this aliquot cannot be divided further if any precipitates are formed, to avoid uneven precipitates in different portions taken from the aliquot. Prior to the urinary analysis, the entire aliquot should be thawed at RT and then diluted (e.g., at least five times).^5^ In this case, warming at 37°C is not necessary. Nevertheless, for most research studies and clinical applications, to immediately divide the urine samples into multiple, small aliquots is not practical. (ii) For frozen urine samples, they can be simply warmed to 37°C (i.e., returned to the body temperature) for 15–20 mins to re‐dissolve any precipitates,^3^, ^4^ which will ensure that any DNA modifications are released from the precipitates. From the practical viewpoint, we propose that warming to 37°C is the most effective, and simplest method to address the issue of adducts associated with precipitates.

**FIGURE 1:**
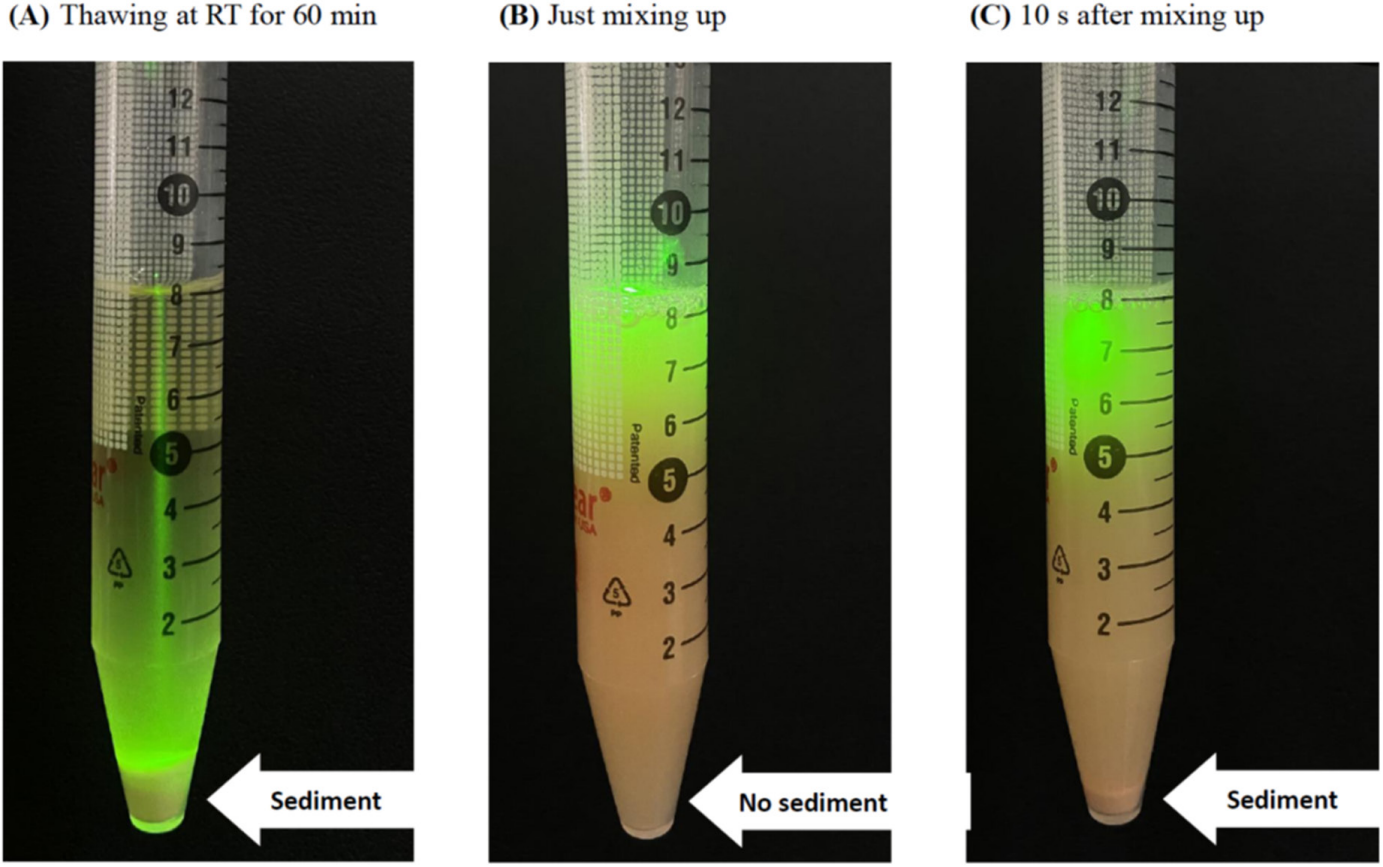
Distribution of precipitates in a representative, frozen urine after thawing at RT for 60 min (A), followed by thorough mixing, using a vortexer, for 5 s and allowed to rest for (B) less than 3 s, and then (C) 10 s. A green‐light laser pointer was used to better demonstrate the distribution of the precipitates.

## AUTHOR CONTRIBUTIONS

Chiung‐Wen Hu and Mu‐Rong Chao: conceptualization, experimental design, writing—original draft preparation, reviewing and editing. Marcus S. Cooke: writing—original draft preparation, reviewing and editing. and Yi‐Jhen Wang: experimental performance.

## FUNDING INFORMATION

The work reported here was supported by the Taiwan Ministry of Science and Technology grant MOST 106‐2314‐B‐040‐015‐MY3 to M‐RC and in part by NIEHS grant R15ES027196 to MSC and C‐WH.

## CONFLICT OF INTEREST STATEMENT

None declared.

## Data Availability

The original experimental data are available on request from the corresponding author.
